# Case of Remarkable Low Temperature

**Published:** 1881-08

**Authors:** 


### Case of Remarkably Low Temperature.

Dr. Walter Mendelson (*New York Medical Record*, June 4, p. 627) gives the case of a man brought into New York Hospital in a starving condition, extremely emaciated, weak, and almost voiceless. The surface was cool, the hands and feet being cold; the heart-sounds were almost inaudible, and the pulse beat forty-three in the minute; the temperature, taken several times and with two different thermometers, in the rectum, was 90.6° F. He was ordered stimulants by the stomach, with a hypodermic injection of equal parts of brandy and ether. Food was given gradually, and the patient slowly recovered strength, the thermometer showing a progressive increase in temperature almost hourly until the normal was reached in about twenty hours. There was a tendency to fall, however, which persisted for some days, the thermometer showing 97.5° in the morning, but always rising to the normal in the afternoon. There was no febrile reaction, the highest temperature being 99.6° on the third day after admission. The digestion seemed in no way disturbed, for three days after admission the patient was eating heartily of everything, and was taking cod-liver oil and iron. He soon regained strength, but appeared to be in a state of mild dementia, which persisted during the two or three weeks of his stay in the hospital. His previous history in respect to mental condition could not be ascertained.

